# Supplementary material for: Theoretical and Experimental Study on Carbodiimide Formation
Source: Int J Mol Sci. 2024 Jul 22;25(14):7991. doi: 10.3390/ijms25147991 (PMC11276972; doi:10.3390/ijms25147991)
Supplement: Supplementary file 1 [file ijms-25-07991-s001.zip › ijms-3113238-supplementary.pdf]

International Journal of
Molecular Sciences

MDPI

Theoretical and Experimental Study on Carbodiimide Formation

Marcell Dániel Csécsi [1,2], Virág Kondor [1], Edina Reizer [1], Renáta Zsanett Boros [3], Péter Tóth [3], László Farkas [3], Béla Fiser [1,2], Zoltán Mucsi [1], Miklós Nagy [1] and Béla Viskolcz [1,*]

1 Institute of Chemistry, University of Miskolc, H-3515 Miskolc, Hungary; marcell.daniel.csecsi@uni-miskolc.hu (M.D.C.); viragkondor@gmail.com (V.K.); edina.reizer@uni-miskolc.hu (E.R.); bela.fiser@uni-miskolc.hu (B.F.); zoltan.mucsi@uni-miskolc.hu (Z.M.); miklos.nagy@uni-miskolc.hu (M.N.)
2 Higher Education and Industrial Cooperation Centre, University of Miskolc, H-3515 Miskolc, Hungary
3 BorsodChem Ltd., Bolyai tér 1, H-3700 Kazincbarcika, Hungary; renata.boros@borsodchem.eu (R.Z.B.); peter.toth45@borsodchem.eu (P.T.); laszlo.farkas@borsodchem.eu (L.F.)
* Correspondence: bela.viskolcz@uni-miskolc.hu

Supporting Information

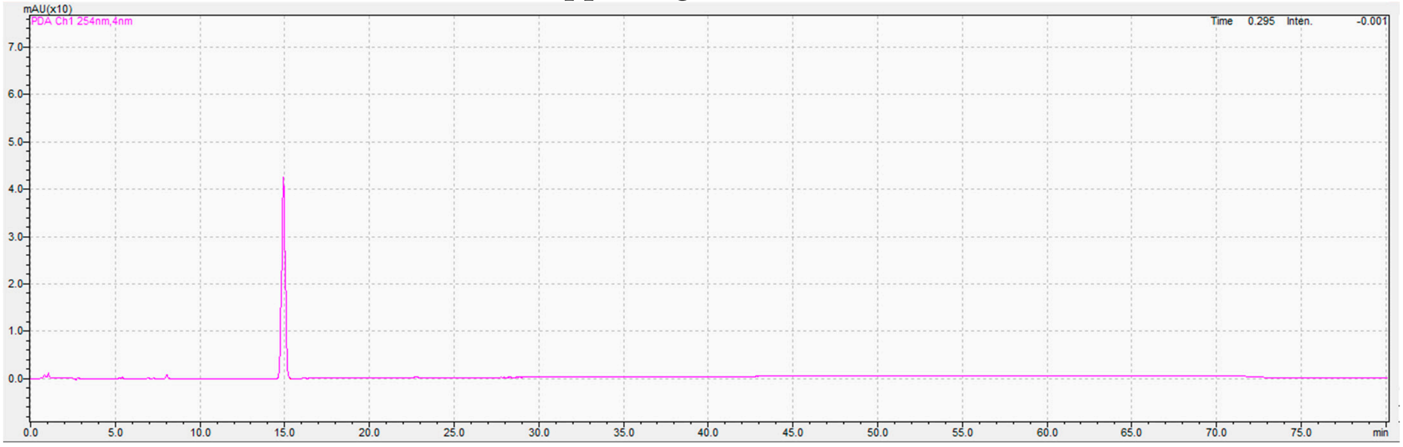

Figure S1. HPLC chromatogram of ODCB used in the experiments as a solvent, 15 min peak indicates the ODCB. Parameters: Shimadzu HPLC, UV detector, 254 nm, Column: Phenomenex, Luna Omega (5 μm, C18, 100 Å, 250 mm x 4.6 mm), Eluent flow rate: 1 mL/min, Column space temperature: 40 °C, Amount of sample injected 1 μL, Eluent: HPLC grade acetonitrile, high purity distilled water. Sample preparation: 0.1 g ODCB + 1.0 g dichloromethane + 2.5 g methanol

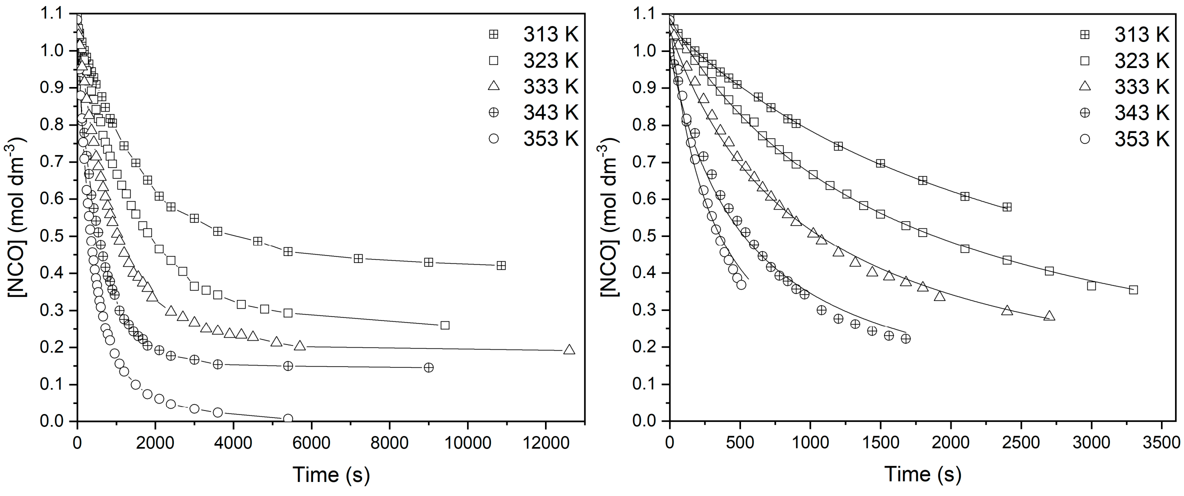

(a) **(b)**

Figure S2. (**a**) Full experimental kinetic diagram. (**b**) Second-order kinetic diagram with nonlinear fits. Fit intervals agree with fit intervals of linear regressions. Calculated rate constants: $k_{(313\ K)}$ = 1.68 · 10^{-4} mol^{-1} dm^3 s^{-1}, $k_{(323\ K)}$ = 2.87 · 10^{-4} mol^{-1} dm^3 s^{-1}, $k_{(333\ K)}$ = 5.17 · 10^{-4} mol^{-1} dm^3 s^{-1}, $k_{(343\ K)}$ = 9.91 · 10^{-4} mol^{-1} dm^3 s^{-1}, $k_{(353\ K)}$ = 17.1 · 10^{-4} mol^{-1} dm^3 s^{-1}.

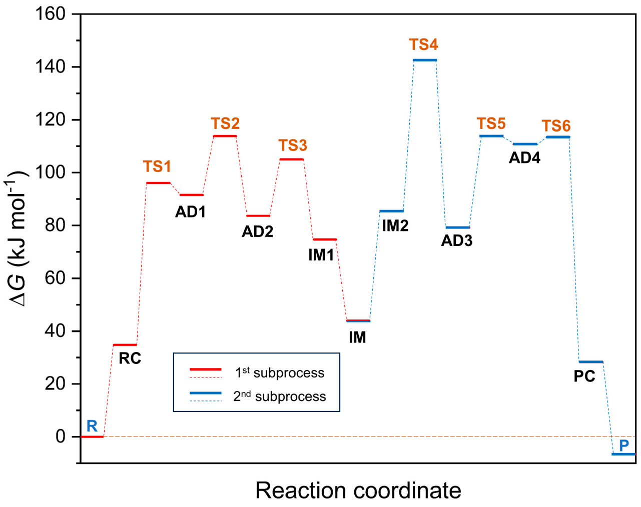

Figure S3. Potential energy surface (PES) of the reaction with the intermediates, complexes and transition states; calculated relative Gibbs free energies (Δ*G*) using B3LYP/6-31G(d) level of theory at 298.15 K and 1 atm in liquid phase, using SMD solvent model in ODCB phase.

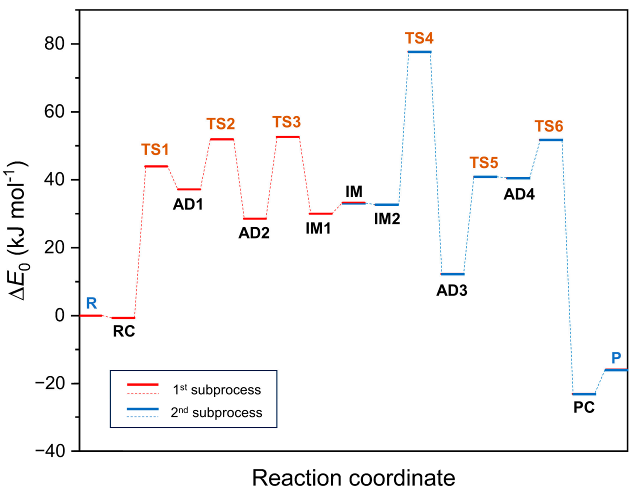

Figure S4. Potential energy surface (PES) of the reaction with the intermediates, complexes and transition states; calculated relative sum of electronic and zero-point energies (ΔE_0) using B3LYP/6-31G(d) level of theory at 298.15 K and 1 atm in liquid phase, using SMD solvent model in ODCB phase.

Table S1. Cartesian coordinates of the stationary points for the reaction system studied. The calculated at the B3LYP/6–31G(d) level of theory in ODCB solvent at 298.15 K and 1 atm. NCO—phenyl isocyanate, MPPO—3-methyl-1-phenyl-2-phospholene-l-oxide catalyst, RC—reactant complex, TS—transition states, AD—intermediate adducts, IM—intermediate product, IM1, IM2—intermediate complexes, PC—product complex, CDI—diphenylcarbodiimide.

NCO			
C	−2.54058700	−0.07089200	0.00013700
N	−1.44913200	−0.58542500	0.00011900
O	−3.66343700	0.29348200	−0.00022300
C	−0.08919800	−0.25192900	0.00004300
C	0.84661300	−1.29328500	0.00001300
C	0.33935400	1.08399100	0.00003700
C	2.20949000	−0.99561700	−0.00002600
H	0.49759800	−2.32115500	0.00002500
C	1.70392200	1.36743000	−0.00000400
H	−0.39528600	1.88407900	0.00008300
C	2.64346300	0.33209900	−0.00003800
H	2.93265800	−1.80647700	−0.00004800
H	2.03251700	2.40311800	−0.00000400
H	3.70559300	0.55976400	−0.00007000

MPPO			
P	−0.46869600	0.91197800	−0.08999500
O	−0.37864100	2.35665200	−0.52640000
C	−1.32794600	0.57517800	1.51631700
C	−2.66693200	−0.10608000	1.15482700
C	−2.62661600	−0.57122800	−0.29064300
C	−1.56771900	−0.17089200	−1.01837200
C	1.18996200	0.13726300	−0.03992700
C	−3.75949100	−1.41095200	−0.80160000
C	1.36382000	−1.23540100	0.19676100
C	2.31505500	0.94663300	−0.24644800
C	2.64525100	−1.78667500	0.23093600
C	3.59745200	0.39385500	−0.21250000
C	3.76341200	−0.97201900	0.02697400
H	−1.46138200	1.51556300	2.05912900
H	−0.70363100	−0.07905400	2.13236500
H	−3.51269700	0.58321100	1.28176600
H	−2.86960900	−0.95715300	1.81718700
H	−1.42989500	−0.40806400	−2.06997800
H	−3.83566500	−2.34588600	−0.23016100
H	−3.64172700	−1.65855700	−1.86061500
H	−4.71576800	−0.88658900	−0.67015900
H	0.49893000	−1.87655600	0.34841200
H	2.17577400	2.00676500	−0.43699400
H	2.77204800	−2.85056100	0.41342800
H	4.46474800	1.02867600	−0.37388300
H	4.76094300	−1.40276100	0.05266800

CO_2			
C	0.00000000	0.00000000	0.00000000
O	0.00000000	0.00000000	1.16889700
O	0.00000000	0.00000000	−1.16889700

RC				TS1			
P	−1.92939700	−0.36324100	−0.68103800	P	1.59645400	−0.37394400	0.58351700
O	−1.24071300	−0.77946500	−1.96074600	O	0.72083600	−0.69973000	1.82344500
C	−3.73364800	−0.76081200	−0.53567600	C	3.35768700	−0.82013800	0.89194600
C	−3.84506000	−1.88556500	0.51792000	C	3.65398500	−2.03087700	−0.02246900
C	−2.53909100	−1.99375300	1.28599400	C	2.52372700	−2.21015200	−1.01701400
C	−1.51005100	−1.26018500	0.82344700	C	1.43588400	−1.43262400	−0.84900800
C	−1.70099100	1.42641800	−0.36345500	C	1.45599300	1.38121700	0.13127800
C	−2.48281900	−2.91043200	2.47197100	C	2.67597600	−3.24080900	−2.09405600
C	−2.14176900	2.03477700	0.82249600	C	1.90247500	1.83890100	−1.11775200
C	−1.06958600	2.20637100	−1.34193600	C	0.93697200	2.29336900	1.06103100
C	−1.95872500	3.40379900	1.02131500	C	1.82963600	3.19673000	−1.43096400
C	−0.88554400	3.57662500	−1.14127300	C	0.86713300	3.65056100	0.74360700
C	−1.33109200	4.17614000	0.03883300	C	1.31280600	4.10272700	−0.50085300
H	−4.11711100	−1.05669500	−1.51659400	H	3.49496400	−1.03981000	1.95397800
H	−4.27970500	0.13363700	−0.22068100	H	3.98969300	0.03643700	0.64072100
H	−4.06040300	−2.85417800	0.04675300	H	3.76009000	−2.95334700	0.56306900
H	−4.67391700	−1.69691500	1.21191200	H	4.60320700	−1.89715100	−0.55520800
H	−0.51534700	−1.26817800	1.26038500	H	0.54836600	−1.47648900	−1.46926100
H	−3.20863800	−2.59941500	3.23547500	H	3.55095500	−3.01583100	−2.71818400
H	−1.48779200	−2.93327500	2.92601300	H	1.79218600	−3.29715500	−2.73555800
H	−2.75641200	−3.93349500	2.18002400	H	2.85274400	−4.23042000	−1.65200400
H	−2.62384300	1.43970700	1.59425600	H	2.30082600	1.13771700	−1.84630200
H	−0.72373300	1.73045600	−2.25482000	H	0.58281300	1.93949200	2.02435100
H	−2.30244700	3.86767600	1.94215900	H	2.17212900	3.54580400	−2.40120200
H	−0.39521800	4.17409200	−1.90536900	H	0.46147100	4.35299600	1.46642100
H	−1.18834100	5.24219600	0.19535100	H	1.25511000	5.15955500	−0.74709000
C	1.79886500	−2.16641100	−1.25942300	C	−1.13602600	−0.75994000	1.68006500
O	1.45092900	−3.10663600	−1.87905000	O	−1.50446100	−1.08987700	2.76485600
N	2.03511800	−1.17442000	−0.61009300	N	−1.35385500	−0.42234900	0.47700100
C	3.12932500	−0.50197300	−0.05159900	C	−2.67488700	−0.36491300	−0.02042000
C	2.89181000	0.71444400	0.60074200	C	−2.83040200	0.11360800	−1.33416400
C	4.43280800	−1.01695400	−0.12900400	C	−3.82408200	−0.75486900	0.69389100
C	3.95584300	1.41125000	1.17348900	C	−4.09328700	0.20016500	−1.91741100
H	1.87882700	1.10159500	0.64779900	H	−1.94347500	0.41973400	−1.88208800
C	5.48720300	−0.31055200	0.44728500	C	−5.08463700	−0.66555000	0.10174900
H	4.60900800	−1.96112500	−0.63693400	H	−3.72762900	−1.12578900	1.70899900
C	5.25530400	0.90369500	1.10040100	C	−5.23066700	−0.18958400	−1.20390300
H	3.76563600	2.35485200	1.67755600	H	−4.18844600	0.57451400	−2.93383400
H	6.49469100	−0.71284400	0.38436600	H	−5.95995100	−0.97207300	0.66966000
H	6.08145700	1.44897300	1.54778800	H	−6.21558900	−0.12266000	−1.65795100
**AD1**				**TS2**			
P	1.48636300	−0.30723100	0.46919800	C	0.91632100	−0.30360700	2.15105700
C	3.23859400	−0.65886400	0.89413400	O	−0.40841700	−0.01242000	2.35540700
C	3.61183300	−1.92228500	0.08676700	O	1.68832300	−0.72900200	2.98515200
C	2.56138300	−2.17124900	−0.97495700	P	−0.76635600	0.50337400	0.69452900
C	1.43995600	−1.42157800	−0.91536700	N	1.04477200	0.01500700	0.82610400
C	1.25582500	1.44161700	0.07534000	C	2.20172300	−0.07677700	0.03401100
C	2.80950900	−3.23684900	−1.99650100	C	3.47699700	0.11335900	0.59702700
C	1.52892300	1.91035800	−1.21766400	C	2.09455500	−0.35244600	−1.33968500
C	0.87701800	2.33970500	1.08427500	C	4.61412200	0.02455700	−0.20507900
C	1.41761800	3.27284400	−1.49879900	H	3.56580400	0.31742200	1.65775600
C	0.76951600	3.69955900	0.79464800	C	3.23905100	−0.41829800	−2.13499600
C	1.03867200	4.16674800	−0.49475000	H	1.11606500	−0.52692400	−1.77610200

H	3.34955500	−0.78439200	1.97390000	C	4.50503500	−0.23330600	−1.57450700
H	3.84453000	0.19930600	0.58936200	H	5.59305100	0.16872700	0.24532500
H	3.66990800	−2.80468600	0.73699400	H	3.13746800	−0.62876800	−3.19664300
H	4.59917200	−1.81636100	−0.37735700	H	5.39487900	−0.29227900	−2.19507400
H	0.59458900	−1.51411600	−1.58460600	C	−0.47809100	1.78970400	−0.55638600
H	3.71485900	−3.00567600	−2.57291200	H	0.42254100	1.82456500	−1.16023900
H	1.96938000	−3.34787600	−2.68705200	C	−2.24354300	1.47601100	1.34674400
H	2.99001100	−4.20028200	−1.50178500	H	−1.90104700	2.00731600	2.24121500
H	1.82161000	1.21839600	−2.00234300	H	−3.04401200	0.79695200	1.65483200
H	0.65847800	1.98016400	2.08518400	C	−2.65892800	2.47125700	0.25911400
H	1.62308200	3.63307900	−2.50281100	H	−3.49664700	2.08165700	−0.33678500
H	0.47153000	4.39293000	1.57585600	H	−2.99873200	3.42725500	0.67692200
H	0.95110400	5.22667300	−0.71683900	C	−1.47425600	2.68669500	−0.66141000
N	−1.14050000	−0.48610600	0.43105200	C	−1.48316000	−0.99185800	−0.08630300
C	−2.46675700	−0.46437500	−0.00796700	C	−1.73125600	−2.14160400	0.68045600
C	−3.59987600	−0.83471900	0.75329500	C	−1.81875000	−0.99048000	−1.44823200
C	−2.68416100	−0.04072700	−1.33834600	C	−2.29825100	−3.27103900	0.08881300
C	−4.87854000	−0.78050100	0.19582700	H	−1.48360800	−2.15434400	1.73691100
H	−3.46263600	−1.15957800	1.77730000	C	−2.38702600	−2.12330800	−2.03554000
C	−3.96409900	0.01073600	−1.88474800	H	−1.63748400	−0.10714800	−2.05306300
H	−1.82186100	0.25453800	−1.93095700	C	−2.62660000	−3.26525000	−1.26923300
C	−5.07663700	−0.36014900	−1.12203800	H	−2.48390200	−4.15593400	0.69150100
H	−5.73207700	−1.07185800	0.80477700	H	−2.64030200	−2.11054200	−3.09215200
H	−4.09371900	0.34380900	−2.91226100	H	−3.06829100	−4.14629600	−1.72685700
H	−6.07655500	−0.32080300	−1.54611700	C	−1.50504000	3.83265800	−1.62583900
C	−0.82646000	−0.77749100	1.67284000	H	−2.39319300	3.76953600	−2.26893200
O	0.67088600	−0.70574400	1.79679100	H	−1.58214600	4.78437100	−1.08255100
O	−1.38298800	−1.07534700	2.71575900	H	−0.61516600	3.86254600	−2.26127300
**AD2**				**TS3**			
P	−0.52108600	0.39519600	0.46178700	P	−0.53439400	0.53785900	0.30544900
O	−0.59560200	−1.12885500	2.24981400	O	−0.31376100	−1.12748600	2.82955300
C	−0.86449500	1.61960400	1.79516300	C	−1.25608600	1.56773400	1.65063000
C	−0.66696000	3.02561100	1.19202200	C	−1.10382800	3.03602300	1.18759600
C	−0.21905700	2.90780300	−0.25000200	C	−0.72778100	3.07700200	−0.27947600
C	−0.09693600	1.66256000	−0.74614000	C	−0.42695600	1.89938300	−0.85995000
C	−1.98012100	−0.47671400	−0.19433100	C	−1.76735800	−0.67295300	−0.29592400
C	0.04162500	4.16521400	−1.02338200	C	−0.69306500	4.40333500	−0.97673600
C	−3.16393600	0.24404600	−0.40467500	C	−1.43479700	−2.03164000	−0.37766700
C	−1.91919300	−1.83287900	−0.54060200	C	−3.04581500	−0.24714800	−0.69128500
C	−4.28195500	−0.39164300	−0.94750300	C	−2.37065100	−2.95520800	−0.84851900
C	−3.03603200	−2.46028800	−1.09326800	C	−3.97841100	−1.17251900	−1.15887200
C	−4.21843600	−1.74305900	−1.29398500	C	−3.64144700	−2.52733200	−1.23744800
H	−0.17056800	1.40582800	2.61051900	H	−0.73544600	1.35310200	2.58409600
H	−1.87361900	1.44786400	2.17717400	H	−2.30685300	1.29380500	1.78143000
H	0.07379400	3.59896100	1.76368900	H	−0.32619200	3.55099400	1.76673500
H	−1.59810000	3.60536400	1.23398900	H	−2.03074700	3.59792100	1.35517100
H	0.18758900	1.45249800	−1.77152800	H	−0.12350800	1.78811700	−1.89515900
H	−0.85499900	4.79901200	−1.04137000	H	−1.68217300	4.87914000	−0.94527100
H	0.34427000	3.95754500	−2.05360300	H	−0.38166400	4.30992500	−2.02088600
H	0.83150900	4.75427000	−0.53851900	H	−0.00116200	5.08508800	−0.46448000
H	−3.21820100	1.29891800	−0.15017800	H	−0.44596200	−2.36503000	−0.07810000
H	−1.00655100	−2.39694800	−0.37737000	H	−3.31292600	0.80527600	−0.64016100
H	−5.19852500	0.17072800	−1.10232100	H	−2.10565400	−4.00708100	−0.90939400
H	−2.98305900	−3.51188400	−1.36098000	H	−4.96585300	−0.83694000	−1.46372400
H	−5.08770400	−2.23620400	−1.72035300	H	−4.36893600	−3.24681700	−1.60334100
C	0.56655700	−1.43477500	1.83238600	C	0.77032300	−1.34901300	2.36966500

O	1.40954400	−2.22869900	2.23542800	O	1.81021600	−1.92930600	2.39406400
N	0.82064100	−0.61352200	0.63661800	N	0.88266300	−0.13849900	0.74655600
C	2.03771600	−0.59782500	−0.10577300	C	1.98267800	−0.27142200	−0.11895800
C	2.03509500	−0.79056000	−1.49338600	C	1.87181300	−0.57710100	−1.49194200
C	3.25531100	−0.42085100	0.56603200	C	3.27903500	−0.11587600	0.41495900
C	3.23626500	−0.77900800	−2.20530600	C	3.00663900	−0.69570500	−2.29676800
H	1.09409800	−0.95798900	−2.00978300	H	0.88869800	−0.73536400	−1.92857000
C	4.45347000	−0.43378600	−0.14794200	C	4.40826900	−0.25347900	−0.38957200
H	3.25272100	−0.28683700	1.64208700	H	3.38031300	0.11584200	1.47018200
C	4.44913300	−0.60434100	−1.53544000	C	4.28339700	−0.53801100	−1.75357100
H	3.22197300	−0.92246900	−3.28247800	H	2.88756600	−0.92433600	−3.35342700
H	5.39315900	−0.30054600	0.38167000	H	5.39451200	−0.12686600	0.05120700
H	5.38418200	−0.60560800	−2.08892000	H	5.16585200	−0.63724900	−2.37984900
**IM1**				**IM**			
P	0.73995300	−0.46621800	0.21935300	P	−0.36684100	0.15426300	0.07308600
O	−0.32652300	1.29624900	3.46066400	C	−0.26949200	1.19505800	1.61574300
C	1.75366300	−0.81366100	1.71616300	C	−0.14414200	2.66683800	1.16235000
C	2.46236500	−2.16322900	1.45459300	C	−0.26113500	2.75760000	−0.34862800
C	2.33104500	−2.53846500	−0.00918300	C	−0.39120800	1.59578200	−1.01430200
C	1.53133500	−1.75878900	−0.76101700	C	−2.00529900	−0.64364600	−0.00722900
C	1.21882800	1.15593400	−0.49942400	C	−0.21318900	4.11449400	−0.98605200
C	3.06758900	−3.74674500	−0.50553300	C	−3.16742100	0.14325400	0.00900000
C	2.45272400	1.33044500	−1.14603800	C	−2.11747600	−2.03877800	−0.06922200
C	0.34348900	2.24530500	−0.38190200	C	−4.42500600	−0.46027000	−0.03172500
C	2.80532400	2.57799500	−1.66274600	C	−3.37708100	−2.63935100	−0.11250700
C	0.69746600	3.49169700	−0.90210700	C	−4.53115000	−1.85247000	−0.09265100
C	1.92793100	3.65929800	−1.54167000	H	0.59039300	0.86418800	2.20520300
H	1.09998000	−0.83475000	2.59002300	H	−1.16634400	1.02408900	2.21754100
H	2.47677100	−0.00420100	1.85220700	H	0.81606200	3.09884300	1.47438000
H	2.02335300	−2.96284200	2.06594400	H	−0.91944300	3.28970200	1.62669600
H	3.52133400	−2.11180800	1.73619800	H	−0.47026700	1.51691000	−2.09512000
H	1.32974800	−1.93571300	−1.81302000	H	−1.02565400	4.74810400	−0.60577200
H	4.14919700	−3.62644100	−0.35799400	H	−0.29526800	4.05901700	−2.07534200
H	2.87843400	−3.93569500	−1.56615200	H	0.72563900	4.62500700	−0.73199400
H	2.77149600	−4.63693200	0.06580300	H	−3.09384300	1.22692200	0.04712100
H	3.13671400	0.49227300	−1.24936200	H	−1.21390800	−2.63892300	−0.08718900
H	−0.61609200	2.11325900	0.10942000	H	−5.32011000	0.15572100	−0.01987700
H	3.76171200	2.70486500	−2.16297800	H	−3.45598100	−3.72208600	−0.16199500
H	0.01148600	4.32943000	−0.81009200	H	−5.51082100	−2.32169600	−0.12645100
H	2.20228100	4.62935600	−1.94757600	N	0.69129000	−1.02570600	−0.14604400
C	−1.44371300	1.31586600	3.11378600	C	2.07422600	−0.94600000	−0.10189700
O	−2.57128900	1.36977000	2.81249100	C	2.79788100	−2.16183700	−0.19464700
N	−0.81874600	−0.58770400	0.58949900	C	2.83915700	0.24063800	0.02580900
C	−1.86986000	−0.68020500	−0.31408800	C	4.18848800	−2.19037000	−0.15224500
C	−1.76264200	−0.58637000	−1.72458200	H	2.23060500	−3.08385500	−0.29703300
C	−3.16897200	−0.89446700	0.21060900	C	4.23388900	0.20331900	0.06869200
C	−2.88268600	−0.70598900	−2.54857600	H	2.33581900	1.20213700	0.07917600
H	−0.79141000	−0.41004700	−2.18120700	C	4.92525900	−1.00737400	−0.01762700
C	−4.28144600	−1.01067500	−0.61768000	H	4.70351500	−3.14612500	−0.22473700
H	−3.27614600	−0.96505700	1.28938700	H	4.78426400	1.13685800	0.16821300
C	−4.15335700	−0.91970500	−2.00888400	H	6.01112400	−1.03013200	0.01602100
H	−2.75596400	−0.62868900	−3.62665600				
H	−5.26038100	−1.17548500	−0.17189900				
H	−5.02259200	−1.01191800	−2.65439500				

IM2				TS4			
P	2.04168200	−0.15316800	−0.35893000	P	1.74945600	−0.10980600	−0.47361600
C	2.38440200	−1.27253000	−1.77860500	C	2.13055800	−0.66006200	−2.18973600
C	3.76862200	−0.85795300	−2.32822700	C	3.29618000	0.23559800	−2.67271600
C	4.47230600	0.03489200	−1.32312800	C	3.88788300	0.98920000	−1.49898000
C	3.74570100	0.43585500	−0.26277900	C	3.22811800	0.89974000	−0.32835000
C	1.69711400	−1.14177500	1.15036100	C	1.87599600	−1.52876500	0.67760900
C	5.89494200	0.42769700	−1.58908600	C	5.13621100	1.79055000	−1.71457300
C	2.72894800	−1.77114100	1.86435600	C	3.04279800	−2.30914300	0.72268000
C	0.37018700	−1.29286300	1.58007300	C	0.80393400	−1.83827500	1.52542600
C	2.43552300	−2.54166500	2.99069700	C	3.13066600	−3.38749200	1.60247300
C	0.08007900	−2.06337700	2.70749800	C	0.89620500	−2.91818400	2.40650300
C	1.11139600	−2.68810200	3.41316200	C	2.05681500	−3.69324200	2.44448300
H	1.57937100	−1.17694900	−2.51022500	H	1.23447200	−0.56848700	−2.80369500
H	2.39528900	−2.30528200	−1.41781300	H	2.41707900	−1.71531400	−2.16707900
H	3.66824400	−0.31200700	−3.27580300	H	2.94892100	0.96109600	−3.41996000
H	4.38464000	−1.73856100	−2.54801400	H	4.07187600	−0.36237400	−3.16649400
H	4.11930900	1.10296300	0.50817100	H	3.52927300	1.40497900	0.58292600
H	6.53759700	−0.46157300	−1.63824100	H	5.95656700	1.14069800	−2.04706100
H	6.28594400	1.09879700	−0.81887400	H	5.44956900	2.31423400	−0.80706200
H	5.97864500	0.92803400	−2.56331500	H	4.98133700	2.53112600	−2.51048400
H	3.76101500	−1.65816900	1.54324700	H	3.88400200	−2.07292900	0.07620000
H	−0.43146100	−0.80509400	1.03298100	H	−0.09810800	−1.23453100	1.49783900
H	3.23992700	−3.02428500	3.53935800	H	4.03608800	−3.98743500	1.63247600
H	−0.95014400	−2.17348000	3.03514200	H	0.06073800	−3.15250100	3.06032500
H	0.88443700	−3.28619600	4.29173100	H	2.12705100	−4.53369000	3.12974200
N	0.86489900	0.86133900	−0.76090400	N	0.32643400	0.67664300	−0.39387200
C	0.46155400	1.98314000	−0.05331700	C	0.09484300	1.76695200	0.45811600
C	1.03682000	2.45518100	1.15343700	C	0.65146200	1.90165300	1.74869500
C	−0.61453700	2.74026600	−0.58269100	C	−0.77036500	2.79010900	0.01224900
C	0.56463800	3.60809800	1.78174500	C	0.37468900	3.01773700	2.54108000
H	1.86249100	1.90932400	1.60456600	H	1.29725700	1.11919600	2.13962300
C	−1.07982800	3.89005300	0.05071500	C	−1.05579200	3.89320100	0.81397000
H	−1.07003200	2.39765500	−1.50838000	H	−1.20620300	2.70277700	−0.97793300
C	−0.49620600	4.33954200	1.24138700	C	−0.48225700	4.02088900	2.08387700
H	1.03518200	3.93640900	2.70647300	H	0.82639900	3.09417400	3.52761000
H	−1.90551400	4.44320900	−0.39249100	H	−1.72423300	4.66510500	0.43917400
H	−0.85931900	5.23753200	1.73379000	H	−0.70168900	4.88539900	2.70463100
C	−1.85000000	−1.21335500	−2.10052100	C	−1.33568700	−0.53240300	−0.97095100
N	−2.54270600	−0.55122000	−1.36772800	N	−2.25978700	0.06661100	−0.39049400
O	−1.07832700	−1.76932800	−2.80056500	O	−0.82702600	−1.37918100	−1.64504400
C	−3.82490200	−0.54070700	−0.80419800	C	−3.61625800	−0.31493800	−0.47766200
C	−4.16189100	0.51467600	0.05190800	C	−4.54422400	0.46329900	0.23295100
C	−4.75627700	−1.55324500	−1.08005200	C	−4.07612500	−1.41322200	−1.22626900
C	−5.43084800	0.55440000	0.62985200	C	−5.90256700	0.15076600	0.19695900
H	−3.42880800	1.28930100	0.25329200	H	−4.17969400	1.31033600	0.80689300
C	−6.02049500	−1.50125700	−0.49539500	C	−5.43718500	−1.71800600	−1.25672400
H	−4.48425200	−2.36737300	−1.74584400	H	−3.36871300	−2.02316800	−1.78025700
C	−6.36353900	−0.45010900	0.36018400	C	−6.35784900	−0.94111500	−0.54773500
H	−5.68883800	1.37507900	1.29343300	H	−6.60750800	0.76377200	0.75296300
H	−6.73931000	−2.28713500	−0.71086900	H	−5.77838900	−2.56988500	−1.83987000
H	−7.35062500	−0.41520900	0.81234200	H	−7.41656100	−1.18389900	−0.57561700

AD3				TS5			
P	1.39789800	−0.27795100	−0.48758400	P	0.94237600	−0.80684900	−0.56451100
C	1.57596800	−0.53414600	−2.30086800	C	1.13221400	−2.44456000	−1.48017300
C	2.86306500	0.20342800	−2.73061400	C	2.48840900	−2.41212800	−2.19407400
C	3.44959500	0.94950100	−1.55023700	C	2.90720600	−0.96460400	−2.34894200
C	2.81679400	0.81259700	−0.36949000	C	2.23236900	−0.07211300	−1.60155000
C	1.73553700	−1.75402100	0.52221600	C	1.54907000	−1.18100100	1.12370300
C	4.68697400	1.76746700	−1.76289800	C	4.03821400	−0.63097700	−3.27217000
C	2.75754200	−2.63041200	0.12807900	C	2.89132900	−0.96212100	1.46609300
C	1.03399600	−1.98974500	1.71178700	C	0.67954200	−1.74071200	2.07301400
C	3.06662700	−3.73949400	0.91669200	C	3.35422900	−1.28831100	2.74301900
C	1.35283000	−3.09667700	2.49902300	C	1.14682700	−2.06331000	3.34765200
C	2.36585500	−3.97309200	2.10223500	C	2.48341600	−1.83708000	3.68601700
H	0.68060200	−0.12447600	−2.77263400	H	0.31978900	−2.49778900	−2.21266700
H	1.59460300	−1.60507000	−2.51503100	H	1.01657000	−3.29103100	−0.79709600
H	2.65916300	0.90551800	−3.54856600	H	2.45650500	−2.90864800	−3.17193400
H	3.60955200	−0.50341600	−3.11503700	H	3.25889700	−2.93516900	−1.60975900
H	3.15214100	1.27087900	0.55457200	H	2.43579100	0.99320100	−1.60771400
H	5.49997700	1.13917300	−2.15020600	H	4.94056400	−1.18987300	−2.99005100
H	5.02452700	2.24802300	−0.84042000	H	4.27310000	0.43723300	−3.27039400
H	4.50496600	2.54437400	−2.51714400	H	3.79184100	−0.93609500	−4.29829500
H	3.31509500	−2.45336000	−0.78761700	H	3.57819400	−0.53852400	0.73924600
H	0.24071600	−1.31662200	2.02015500	H	−0.35919400	−1.92693500	1.81887000
H	3.85539000	−4.41801200	0.60429900	H	4.39585800	−1.11224900	2.99746600
H	0.80462800	−3.27603100	3.41959700	H	0.46417200	−2.49289100	4.07571900
H	2.60892700	−4.83635700	2.71557400	H	2.84418100	−2.08979000	4.67933800
N	0.05011100	0.58134600	0.05937900	N	0.06481400	0.86257600	−0.23498500
C	0.08132100	1.90156100	0.61914600	C	0.37560100	2.21491700	−0.03336800
C	0.56693400	2.10336600	1.91539500	C	−0.52097900	3.24212700	−0.38752800
C	−0.37870600	2.99094500	−0.13103500	C	1.62321400	2.56455500	0.51500200
C	0.60332100	3.39134600	2.45537600	C	−0.16872200	4.57696400	−0.19092400
H	0.90994500	1.25197000	2.49630600	H	−1.48803600	2.98511900	−0.80204000
C	−0.35433400	4.27278500	0.41773500	C	1.96927900	3.90412700	0.69060400
H	−0.75933700	2.82513600	−1.13381100	H	2.31155700	1.78233900	0.81650700
C	0.14070800	4.47717900	1.70951500	C	1.07675400	4.92045200	0.34197500
H	0.98519600	3.54154900	3.46154100	H	−0.87561000	5.35579800	−0.46643900
H	−0.71641700	5.11425500	−0.16677200	H	2.93923500	4.15059300	1.11527300
H	0.16314500	5.47805100	2.13194600	H	1.34535500	5.96320600	0.48670200
C	−1.14485500	−0.16044100	−0.29209200	C	−1.15632900	0.26012900	−0.25984100
N	−2.28298000	0.39068400	0.03856900	N	−2.32236600	0.74887200	−0.05773400
O	−0.80870000	−1.24209400	−0.86697700	O	−0.79894900	−1.04479400	−0.52131000
C	−3.50829500	−0.21549300	−0.24099600	C	−3.51137400	0.00648300	−0.11895600
C	−4.65841700	0.50779600	0.15515000	C	−4.59728100	0.50520200	0.62854900
C	−3.71982300	−1.46726000	−0.87096400	C	−3.72447800	−1.14313500	−0.90896300
C	−5.94377600	0.01893500	−0.06196400	C	−5.83784300	−0.12886500	0.61105500
H	−4.50870900	1.46919700	0.64050300	H	−4.44093100	1.40004200	1.22514000
C	−5.01255500	−1.94983600	−1.08444600	C	−4.97245400	−1.76749700	−0.92859000
H	−2.86017700	−2.04606200	−1.18427400	H	−2.91396900	−1.54189100	−1.50803000
C	−6.13507700	−1.21869100	−0.68649600	C	−6.03555800	−1.27252300	−0.16853800
H	−6.80167400	0.60686400	0.25780100	H	−6.65453600	0.27540600	1.20442300
H	−5.14149100	−2.91517400	−1.57072600	H	−5.11308800	−2.65081600	−1.54747400
H	−7.13692400	−1.60419600	−0.85760800	H	−7.00334900	−1.76638700	−0.18799900

AD4				TS6			
P	0.84390900	−0.99308400	−0.36454500	P	−1.05785200	−1.41722600	0.09260300
C	0.99382700	−2.84601900	−0.70039000	C	−1.28407200	−3.23162700	0.32482900
C	1.96438200	−3.02538700	−1.88150500	C	−1.52611000	−3.43542100	1.83753500
C	2.21263100	−1.68001400	−2.52578800	C	−1.79128800	−2.09691000	2.49725800
C	1.72485800	−0.60287600	−1.88119800	C	−1.61468700	−0.99687800	1.73820400
C	1.79748000	−0.81763200	1.19231700	C	−2.19041000	−0.79985200	−1.18593400
C	2.99140000	−1.62220700	−3.80378700	C	−2.20358800	−2.07888800	3.93757200
C	3.12554800	−1.27078800	1.21790200	C	−3.54673700	−0.58644300	−0.89711000
C	1.22536100	−0.29608600	2.36021500	C	−1.71427000	−0.59182700	−2.48862400
C	3.87349100	−1.19019200	2.39339700	C	−4.41629900	−0.16460700	−1.90382500
C	1.97089300	−0.23615100	3.53910300	C	−2.58877500	−0.17228000	−3.49165500
C	3.29585500	−0.67701400	3.55705200	C	−3.93841300	0.04192700	−3.20048700
H	−0.00167700	−3.21354800	−0.96460800	H	−0.40312400	−3.76154300	−0.04632700
H	1.31523000	−3.38706400	0.19323400	H	−2.14618800	−3.55533400	−0.26547100
H	1.58046000	−3.73194800	−2.62840600	H	−0.65616800	−3.90070700	2.31900100
H	2.92952200	−3.43284400	−1.55005800	H	−2.37019300	−4.11327000	2.01244200
H	1.84583600	0.41776800	−2.22645200	H	−1.73921500	0.01901700	2.09317700
H	3.97452800	−2.09381100	−3.67383000	H	−3.14583500	−2.62641200	4.07216400
H	3.13733500	−0.59637400	−4.15339000	H	−2.33208100	−1.06065000	4.31478000
H	2.47370100	−2.19136800	−4.58753600	H	−1.45321300	−2.59036700	4.55495300
H	3.58218200	−1.67881500	0.32032100	H	−3.92242800	−0.74271000	0.11045900
H	0.20332400	0.06746100	2.35784400	H	−0.66406000	−0.74980600	−2.71390200
H	4.90495400	−1.53153900	2.39881500	H	−5.46451600	0.00585100	−1.67443800
H	1.51542100	0.16367700	4.44079000	H	−2.21495800	−0.00964100	−4.49869000
H	3.87648200	−0.62000200	4.47367700	H	−4.61673600	0.37186700	−3.98263400
N	0.03424100	0.83166100	−0.25198800	N	0.00259400	1.27843700	0.17121800
C	0.37871000	2.18165700	−0.32484200	C	0.04239800	2.64219600	0.49811800
C	−0.57499600	3.21441300	−0.44693800	C	1.21207100	3.42692200	0.56566200
C	1.74472100	2.53062700	−0.28100300	C	−1.19368900	3.26100200	0.77521700
C	−0.16180200	4.54431800	−0.52670000	C	1.13542300	4.77849100	0.90503500
H	−1.62708200	2.95982200	−0.47330600	H	2.17298000	2.97307600	0.34871000
C	2.14091000	3.86407000	−0.36643300	C	−1.25973500	4.61098600	1.11159400
H	2.49277800	1.75211800	−0.17019600	H	−2.09721600	2.66049700	0.71202200
C	1.19308000	4.88362800	−0.49012800	C	−0.09452700	5.38187900	1.18048400
H	−0.91434100	5.32400700	−0.62006000	H	2.05013200	5.36517100	0.95225300
H	3.20054800	4.10524100	−0.32988000	H	−2.22635400	5.06397100	1.31920700
H	1.50464700	5.92256800	−0.55346900	H	−0.14510900	6.43521900	1.44271900
C	−1.18161000	0.25753100	−0.14328300	C	1.03581700	0.60225800	−0.13733500
N	−2.36319800	0.75008700	−0.07291300	N	2.24517100	0.64977200	−0.46328800
O	−0.84628800	−1.10209000	−0.15849300	O	0.42731000	−1.11252200	−0.27325000
C	−3.52779100	−0.01284100	0.09950800	C	3.29696200	−0.25630900	−0.47232300
C	−4.71431400	0.50998800	−0.45295400	C	4.40227900	0.04482400	−1.29254800
C	−3.61482500	−1.20950600	0.84146000	C	3.34996200	−1.41696300	0.32692400
C	−5.93315300	−0.14694100	−0.29657700	C	5.51596300	−0.79216900	−1.32529000
H	−4.65593200	1.44107600	−1.01049100	H	4.36645100	0.94454400	−1.90065200
C	−4.84035400	−1.85771400	1.00074000	C	4.46956400	−2.24699100	0.28703800
H	−2.72384500	−1.62576300	1.29794300	H	2.51069800	−1.65862700	0.96812400
C	−6.00610100	−1.33819800	0.43171900	C	5.55852400	−1.94564100	−0.53667700
H	−6.83052100	0.27686800	−0.74111400	H	6.35512300	−0.53990200	−1.96902600
H	−4.88182300	−2.77759800	1.57958700	H	4.49133400	−3.13753600	0.91107200
H	−6.95656700	−1.84920200	0.55967900	H	6.42743600	−2.59767400	−0.56047200

PC				CDI			
P	−1.34819600	−1.39016300	0.52368300	N	1.13085100	−1.01944900	−0.45574800
C	−2.08264500	−2.87130300	1.35773100	N	−1.13085200	−1.01954300	0.45551600
C	−2.69826200	−2.35948900	2.67942000	C	2.35066900	−0.38155900	−0.16763500
C	−2.79687200	−0.84428900	2.64133200	C	−2.35066800	−0.38158700	0.16754100
C	−2.20313300	−0.23061600	1.60095600	C	2.48756500	0.57661400	0.85007500
C	−2.05293400	−1.24917800	−1.15950600	C	−2.48754100	0.57690600	−0.84987000
C	−3.51855300	−0.14820900	3.75665000	C	3.46308000	−0.73885300	−0.94173500
C	−1.18367300	−1.27757100	−2.25844300	C	−3.46310400	−0.73914300	0.94148500
C	−3.43323800	−1.11618000	−1.37671200	C	3.72896100	1.16610400	1.08253300
C	−1.68639600	−1.17621100	−3.55784100	C	−3.72893700	1.16645000	−1.08218800
C	−3.93446500	−1.01586000	−2.67515900	C	4.70074500	−0.14259800	−0.70091500
C	−3.06127200	−1.04584100	−3.76695300	C	−4.70076900	−0.14283200	0.70080700
H	−1.30271600	−3.62246000	1.51290400	C	4.83958600	0.81096300	0.31059700
H	−2.84539100	−3.31189900	0.70851200	C	−4.83958600	0.81105100	−0.31040600
H	−2.08850900	−2.65361700	3.54433600	C	0.00000100	−0.91187700	−0.00010100
H	−3.69104100	−2.79544300	2.84775100	H	1.62603100	0.85344500	1.45094500
H	−2.17427400	0.84693100	1.46607100	H	−1.62599100	0.85394300	−1.45062400
H	−4.56328300	−0.48295600	3.80937400	H	3.34256000	−1.48186900	−1.72437600
H	−3.50580500	0.93903800	3.63748600	H	−3.34260400	−1.48240500	1.72389400
H	−3.06161700	−0.39843000	4.72376700	H	3.82781200	1.90679000	1.87168700
H	−0.11575600	−1.37497100	−2.08644000	H	−3.82776600	1.90738900	−1.87110900
H	−4.11759500	−1.08376400	−0.53253800	H	5.55785900	−0.42558100	−1.30589800
H	−1.00547300	−1.19893700	−4.40462200	H	−5.55790100	−0.42601900	1.30566800
H	−5.00421600	−0.91083600	−2.83562200	H	5.80422900	1.27464000	0.49679500
H	−3.45280000	−0.96608200	−4.77768100	H	−5.80422900	1.27477400	−0.49649100
N	1.87339600	2.61135000	0.80605800				
C	0.64697300	3.02846800	0.26220100				
C	0.15261300	2.54848800	−0.96210600				
C	−0.10352200	3.95588700	0.99850000				
C	−1.07887600	2.99765400	−1.43636000				
H	0.72843400	1.82327500	−1.52929400				
C	−1.33255800	4.40254100	0.51276800				
H	0.28865300	4.31678100	1.94457000				
C	−1.82596300	3.92631200	−0.70478100				
H	−1.45584000	2.61630700	−2.38153100				
H	−1.90511500	5.12424100	1.08917800				
H	−2.78466700	4.27307200	−1.08010600				
C	2.78026100	1.90622500	0.38061200				
N	3.82478800	1.36472600	0.05032700				
O	0.16327100	−1.32023300	0.48196800				
C	4.27986100	0.03414700	0.02529800				
C	5.64657000	−0.17017900	−0.21133800				
C	3.42459200	−1.06546800	0.20635200				
C	6.16055600	−1.46593700	−0.25433300				
H	6.29094400	0.69205600	−0.35522700				
C	3.95273900	−2.35545500	0.15651500				
H	2.35966000	−0.92082400	0.37267300				
C	5.31658700	−2.56409900	−0.07038400				
H	7.22182800	−1.61557900	−0.43411500				
H	3.28704600	−3.20351100	0.29540300				
H	5.71668700	−3.57364000	−0.10704800				
